# Race‐specific prostate cancer outcomes in a cohort of low and favorable‐intermediate risk patients who underwent external beam radiation therapy from 1990 to 2017

**DOI:** 10.1002/cam4.4802

**Published:** 2022-05-26

**Authors:** Sean P. Stroup, Audry H. Robertson, Kayla C. Onofaro, Michael G. Santomauro, Nicholas R. Rocco, Huai‐ching Kuo, Avinash R. Chaurasia, Samantha Streicher, Darryl Nousome, Timothy C. Brand, John E. Musser, Christopher R. Porter, Inger L. Rosner, Gregory T. Chesnut, Anthony D'Amico, Grace Lu‐Yao, Jennifer Cullen

**Affiliations:** ^1^ Center for Prostate Disease Research, Murtha Cancer Center Research Program, Department of Surgery Uniformed Services University of the Health Sciences Bethesda Maryland USA; ^2^ Department of Urology Naval Medical Center San Diego San Diego California USA; ^3^ The Henry M. Jackson Foundation for the Advancement of Military Medicine, Inc Bethesda Maryland USA; ^4^ Department of Radiation Oncology Walter Reed National Military Medical Center Bethesda Maryland USA; ^5^ Madigan Army Medical Center Tacoma Washington USA; ^6^ Tripler Army Medical Center Honolulu Hawaii USA; ^7^ Virginia Mason Medical Center Seattle Washington USA; ^8^ Urology Service, Department of Surgery Walter Reed National Military Medical Center Bethesda Maryland USA; ^9^ Department of Radiation Oncology Brigham and Women's Hospital and Dana Farber Cancer Institute, Harvard Medical School Boston Massachusetts USA; ^10^ Department of Medical Oncology Sidney Kimmel Cancer Center at Jefferson, Sidney Kimmel Medical College Philadelphia Pennsylvania USA; ^11^ Sidney Kimmel Cancer Center at Jefferson Philadelphia Pennsylvania USA; ^12^ Philadelphia Jefferson College of Population Health Pennsylvania USA; ^13^ Department of Population and Quantitative Health Sciences Case Western Reserve University Cleveland Ohio USA; ^14^ Case Comprehensive Cancer Center Cleveland Ohio USA; ^15^ Infectious Disease Clinical Research Program Uniformed Services University of the Health Sciences Bethesda Maryland USA; ^16^ Frederick National Laboratory for Cancer Research National Cancer Institute Frederick Maryland USA; ^17^ INOVA Falls Church Virginia USA

**Keywords:** prostatic cancer, radiation therapy, survival, urological oncology

## Abstract

**Background:**

Previous research exploring the role of race on prostate cancer (PCa) outcomes has demonstrated greater rates of disease progression and poorer overall survival for African American (AA) compared to Caucasian American (CA) men. The current study examines self‐reported race as a predictor of long‐term PCa outcomes in patients with low and favorable‐intermediate risk disease treated with external beam radiation therapy (EBRT).

**Methods:**

This retrospective cohort study examined patients who were consented to enrollment in the Center for Prostate Disease Research Multicenter National Database between January 01, 1990 and December 31, 2017. Men self‐reporting as AA or CA who underwent EBRT for newly diagnosed National Comprehensive Cancer Network‐defined low or favorable‐intermediate risk PCa were included. Dependent study outcomes included: biochemical recurrence‐free survival, (ii) distant metastasis‐free survival, and (iii) overall survival. Each outcome was modeled as a time‐to‐event endpoint using race‐stratified Kaplan–Meier estimation curves and multivariable Cox proportional hazards analysis.

**Results:**

Of 840 men included in this study, 268 (32%) were AA and 572 (68%) were CA. The frequency of biochemical recurrence, distant metastasis, and deaths from any cause was 151 (18.7%), 29 (3.5%), and 333 (39.6%), respectively. AA men had a significantly younger median age at time of EBRT and slightly higher biopsy Gleason scores. Multivariable Cox proportional hazards analyses demonstrated no racial differences in any of the study endpoints.

**Conclusions:**

These findings reveal no racial disparity in PCa outcomes for AA compared to CA men, in a long‐standing, longitudinal cohort of patients with comparable access to cancer care.

## Graphical abstract

This study explored whether differences in long‐term oncological outcomes exist for African American compared to Caucasian American men diagnosed with low risk or favorable‐intermediate risk prostate cancer and treated with external beam radiation therapy, in equal access health care settings. Over a 25+ year study period, this study found no significant racial differences in biochemical recurrence‐free, distant metastasis‐free, and overall survival, controlling for detailed clinical factors. These findings suggest that access to, and receipt of, cancer care in equal access health care settings may reduce the long‐reported racial disparities in prostate cancer outcomes.

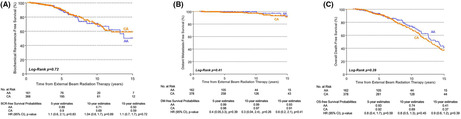

## 1 INTRODUCTION

Prostate cancer (PCa) is the most commonly diagnosed cancer in men and is the second leading cause of cancer‐related death in the United States (U.S.), with an estimated 191,930 new cases and 33,330 deaths projected in 2020.^1^ Despite advances in treatment techniques over the last three decades, African American (AA) men are more likely to present with distant metastasis (DM) at diagnosis and have a 2.5‐fold greater risk of PCa death compared to Caucasian American (CA) men.^2^ Racial differences in cancer outcomes may result from a complex combination of biological factors, access to healthcare, and patterns of cancer care including treatment quality or intensity, and/or other social determinants of health.^3^, ^4^, ^5^, ^6^, ^7^, ^8^, ^9^, ^10^, ^11^ Racial disparities in PCa outcomes continue to be a significant concern and a primary focus of national research.^12^

Temporal changes in approaches to external beam radiation therapy (EBRT) for PCa have made it possible for practitioners to administer more effective treatment with more favorable oncological outcomes.^9^, ^13^, ^14^, ^15^ These changes have resulted in greater doses to the prostate and less dose delivery to nontarget organs.^16^, ^17^, ^18^, ^19^ The introduction of three‐dimensional conformal radiation therapy (3D CRT) in the 1990's allowed for the delivery of higher total fractionated radiation doses.^14^ Subsequent development of intensity‐modulated radiation therapy (IMRT) led to further improvements in cancer outcomes, with further reductions in radiation exposure to nontarget organs.^9^, ^13^ These advancements in treatment delivery have allowed for higher dosing and expanded field size, with increasingly favorable oncological outcomes.^14^, ^15^, ^16^, ^17^, ^20^ Whether such advancements over the last 25+ years have had an impact on race‐specific PCa outcomes has not been adequately addressed.

The primary aim of this study was to examine whether patient self‐reported race was predictive of three outcomes: (i) biochemical recurrence‐free survival (BRFS), (ii) distant metastasis‐free survival (DMFS), and (iii) overall survival (OS) of men with newly diagnosed low and favorable‐intermediate risk PCa whose primary treatment was EBRT. A secondary study aim was to assess whether there was an independent association of EBRT technique on these three study outcomes, given the extended study period. In examining these aims, there was an ability to control for detailed clinical and follow‐up information amassed over the 25+ year study period.

## 2 MATERIALS AND METHODS

### 2.1 Study design, population, and period

This retrospective cohort study examined patients who provided written informed consent to enroll in the Center for Prostate Disease Research (CPDR) Multicenter National Database between January 01, 1990 and December 31, 2017 at one of five participating CPDR sites, including: Walter Reed National Military Medical Center, Madigan Army Medical Center, Naval Medical Center San Diego, Tripler Army Medical Center, and Virginia Mason Medical Center. Stipends were not given for research participation. Institutional Review Board approval for this study was provided by the Uniformed Services University of the Health Sciences. This study utilized de‐identified data that are not publicly available and are protected by privacy safeguards.

Study eligibility was restricted to men with newly diagnosed, biopsy‐confirmed, National Comprehensive Cancer Network (NCCN)‐defined low and favorable‐intermediate risk PCa,^21^ treated with EBRT within 12 months of PCa diagnosis. Patients were also excluded if they had less than 1‐year of follow‐up, M+ or N+ disease at diagnosis. Only those who self‐reported as AA or CA were included, due to limited sample sizes for other racial/ethnic groups. Finally, only patients treated with a total fractionated EBRT dose between 6500 and 8200 centi‐Grays (cGy) were included.

### 2.2 Demographic, clinical, and treatment‐related variables

Detailed patient information that was examined in this study included: patient age at time of initiating EBRT (years), self‐reported patient race (African American and Caucasian American) follow‐up time after EBRT (years), time from initiating EBRT to prostate‐specific antigen (PSA) nadir (years), time from initiating EBRT to biochemical recurrence (BCR) (years), time from initiating EBRT to DM (years), EBRT dose (cGy), prostate gland volume (cubic centimeters, cc), receipt of hormone treatment (HT) (yes vs. no), EBRT technique (3D CRT, IMRT), receipt of secondary HT after EBRT (yes vs. no), PSA nadir value (<0.2 ng/ml vs. ≥0.2 ng/ml), time to nadir (TTN) (<2.3 years vs. ≥2.3 years), PSA at diagnosis (ng/ml), PSA doubling time (PSADT) (months), clinical T stage, biopsy Gleason sum, body mass index (BMI) (kg/m^2^), and number of major comorbidities at diagnosis, defined as presence of the following conditions at time of diagnosis: chronic obstructive pulmonary disease (COPD), cardiovascular disease (CVD), cerebral vascular accident (CVA), and/or other cancer(s).

PSA nadir was defined as the lowest PSA value following EBRT but prior to secondary HT, if applicable.^22^ HT was defined as any HT administered 9 months prior to, or 1 year following, EBRT initiation. Secondary HT was defined as HT administered >1 year following EBRT, and before distant metastasis. PSADT was calculated among those who experienced a BCR event using all PSA values within 2 years after failure, censored at the time of metastasis or secondary HT. PSADT was computed as the natural logarithm of 2 divided by the slope obtained from fitting a linear regression of the natural log (PSA) over time. TTN was dichotomized using a median split (in years) of time from EBRT to PSA nadir. PSADT was categorized as <10 months versus ≥10 months.^23^

### 2.3 Measurement of study endpoints (BRFS, DMFS, and OS)

BCR was defined according to the RTOG‐ASTRO Phoenix Consensus guidelines, as a rise in PSA ≥ 2 ng/ml above the nadir PSA.^24^ DM was confirmed by a positive bone biopsy, bone scan, computed tomography (CT) scan, and/or magnetic resonance imaging (MRI). OS was modeled as the time from EBRT initiation to date of death due to any cause. For all three time‐dependent outcomes, time to event was calculated starting at date of EBRT initiation to the end of study period if no event was observed, censored on the date of achievement of the study endpoint, or censored at last known follow‐up date. Patient vital status was confirmed for all study subjects as part of ongoing follow‐up.

### 2.4 Statistical analysis

Demographic, clinical, treatment, and outcome characteristics were calculated for the overall study cohort and compared across race using the Mann–Whitney test for continuous variables, and chi‐squared tests for categorical variables. Race‐stratified Kaplan–Meier (KM) estimation curves were used to produce 5‐, 10‐, and 15‐year BRFS, DMFS, and OS probability estimates for low risk and favorable‐intermediate risk patients, separately. Multivariable Cox proportional hazards (PH) analyses were used to examine BRFS, DMFS, and OS as a function of race, controlling for key clinical covariates. Hazard Ratios (HR) are reported for Cox PH regression models, with corresponding 95% confidence intervals (CI) and *P*‐values (summary alpha error = 0.05, two‐sided testing). The threshold of *P* < 0.05 was used to define statistical significance. All statistical analyses were conducted using SAS version 9.4 (Cary, North Carolina).

## 3 RESULTS

There were 840 men who met the study inclusion criteria. The median age (interquartile range, IQR) and follow‐up time were 69.9 (IQR = 63.8–74.9) years, and 6.5 (IQR = 4.3–10.9) years, respectively (Table 1). A total of 572 (68%) CA and 268 (32%) AA men composed the study cohort. There were 151 (18.7%) BCR events, 29 (3.5%) DM events, and 333 (39.6%) deaths due to any cause.

**TABLE 1:** Overall and race‐stratified descriptive characteristics of study cohort

	Overall cohort *N* = 840	Caucasian American patients *n* = 572 (68%)	African American patients *n* = 268 (32%)	*p*‐value
	*N* (%)	*N* (%)	*N* (%)	
Age at EBRT, (years)				**<0.001**
Median (IQR^a^)	69.9 (63.8, 74.9)	70.7 (64.7, 75.5)	68.4 (62, 73)	
Follow‐up time after EBRT, (years)				0.13
Median (IQR)	6.5 (4.3, 10.9)	6.9 (4.4, 11.2)	5.9 (4.2, 10.3)	
Time from EBRT to PSA Nadir^b^, (years)				0.17
Median (IQR)	2.3 (1.3, 4.3)	2.2 (1.3, 4.3)	2.6 (1.4, 4.3)	
Time from EBRT to BCR^c^, (years)				0.95
Median (IQR)	5.3 (3.4, 7.8)	5.4 (3.4, 8)	5 (3.6, 7.5)	
Time from EBRT to metastasis, (years)				0.84
Median (IQR)	8.1 (3.1, 11)	8.1 (3, 11.2)	7.1 (3.1, 10.3)	
EBRT dosage (centiGrays)				
Median (IQR) for 3D CRT	7000 (6840, 7040)	7000 (6840, 7040)	7000 (6840, 7020)	0.70
Median (IQR) for IMRT	7800 (7600, 7800)	7800 (7600, 7800)	7800 (7600, 7800)	0.37
Prostate gland volume (cc)				0.80
Median (IQR)	36.5 (28.4, 48.1)	36.8 (28, 48.1)	36 (29.7, 48.2)	
Primary treatment type, *N* (%)				0.53
EBRT alone	748 (89)	512 (89.5)	236 (88.1)	
EBRT with HT^d^	92 (11)	60 (10.5)	32 (11.9)	
Primary EBRT technique, *N* (%)				**<0.001**
3D CRT	530 (63.1)	394 (68.9)	136 (50.7)	
IMRT	310 (36.9)	178 (31.1)	132 (49.3)	
Secondary HT^e^, *N* (%)	68 (8.1)	52 (9.1)	16 (6)	
Time from EBRT to secondary HT, (years) Median (IQR)	6.2 (3.8, 9.4)	6.2 (3.8, 9)	7.6 (3.6, 10.3)	0.73
PSA Nadir (ng/ml), *N* (%)				
<0.2 (undetectable)	241 (29.9)	165 (30)	76 (29.6)	0.90
≥0.2 (detectable)	566 (70.1)	385 (70)	181 (70.4)	
PSA at diagnosis (ng/ml), *N* (%)				
Median (IQR)	5.9 (4.3, 8.3)	5.8 (4.2, 8.2)	6.2 (4.5, 8.5)	0.099
PSADT^g^ (months), *N* (%)				0.92
<10	52 (12.3)	36 (6.6)	16 (6.2)	
≥10	99 (6.4)	69 (12.5)	30 (11.7)	
Clinical T stage, *N* (%)				0.20
≤T2a	758 (90.2)	511 (89.3)	247 (92.2)	
T2b–T2c	82 (9.8)	61 (10.7)	21 (7.8)	
Biopsy Gleason score, *N* (%)				**0.009**
≤6	691 (82.3)	484 (84.6)	207 (77.2)	
3 + 4	149 (17.7)	88 (15.4)	61 (22.8)	
Obese (BMI ≥ 30.0 kg/m^2^), *N* (%)	193 (26.5)	111 (22.1)	82 (36.4)	**<0.001**
Any major comorbidity^f^, *N* (%)	321 (38.2)	254 (44.4)	67 (25.0)	**<0.001**

^a^ IQR, interquartile range.

^b^ PSA nadir was defined as the lowest absolute PSA value following EBRT treatment *and* prior to secondary HT, if applicable.

^c^ BCR, biochemical recurrence, defined as a rise in PSA ≥2 ng/mL above the nadir PSA value.

^d^ HT, hormone treatment, defined as any HT within 9 months prior to *or* 1 year following EBRT treatment (start date).

^e^ Secondary HT was defined as HT >1 year following EBRT and before distant metastasis.

^f^ Major comorbid conditions included: chronic obstructive pulmonary disease (COPD), cardiovascular disease (CVD), Cerebral Vascular Accident (CVA), and/or other cancer(s).

^g^ PSADT, PSA doubling time was calculated among those who experienced a BCR event (*n* = 151) using all PSA values within 2 years after BCR, censored at the time of metastasis or use of secondary HT. PSADT was then computed as the natural logarithm of 2 divided by the slope obtained from fitting a linear regression of the natural log(PSA)/time.

Bold indicates significant values of *p* < 0.05.

Comparisons across race revealed significantly younger median age at EBRT for AA versus CA men (68.4 [IQR = 62–73] versus 70.7 [IQR = 64.7–75.5] years, respectively; *p* < 0.001). A greater proportion of AA men received IMRT (49.3% vs. 31.1%, respectively; *p* < 0.001), while a greater proportion of CA men received 3D CRT (68.9% vs. 50.7%, respectively; *p* < 0.001). Use of secondary HT was very low (8.1%) and did not differ across race.

AA men were also more likely to have a biopsy Gleason score 3 + 4 disease compared to CA men (22.8% vs. 15.4%, respectively; *p* = 0.009), and to be obese (36.4% vs. 22.1%, respectively; *p* < 0.001). CA men were more likely to have major comorbidities than AA men (44.4% vs. 25%, respectively; *p* < 0.001) due mostly to a greater prevalence of “other cancers.”

**FIGURE 1:**
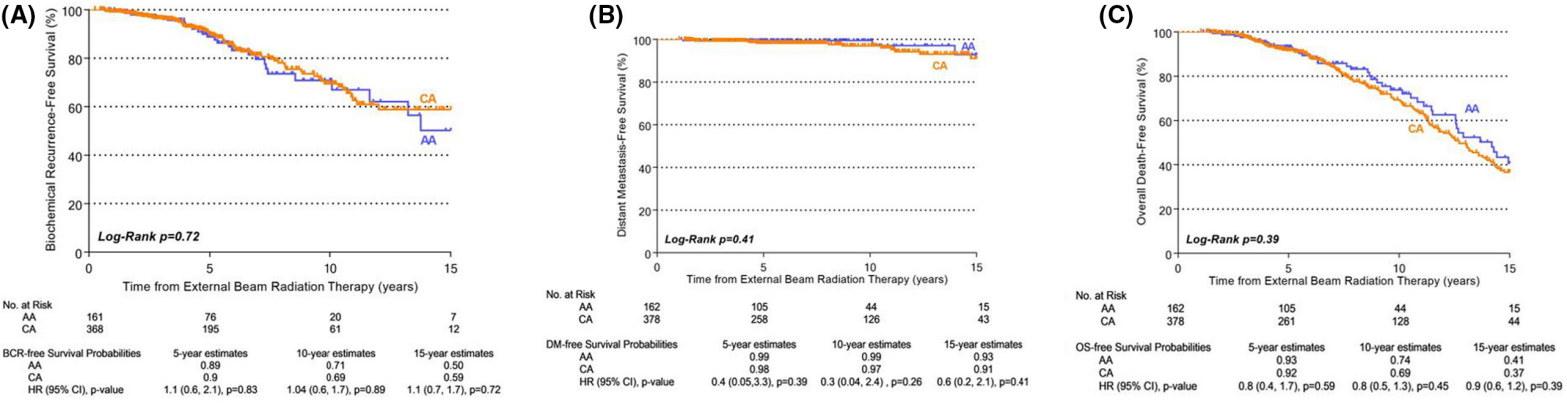
Race‐stratified biochemical recurrence‐free (A), distant metastasis‐free (B), and overall survival (C) in low‐risk patients. Study outcomes were modeled as time‐to‐event endpoints using race‐stratified Kaplan–Meier estimation curves. Low and favorable‐intermediate risk strata were defined by National Comprehensive Cancer Network criteria.^21^ AA, African American; CA, Caucasian American; BCR, biochemical recurrence; DM, distant metastasis; OS, overall survival

**FIGURE 2:**
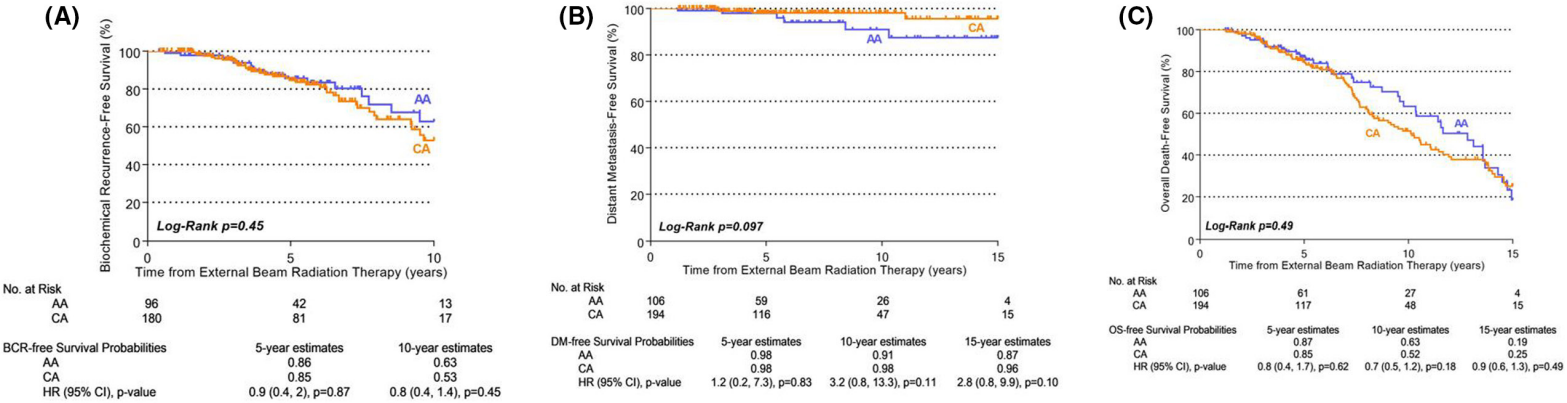
Race‐stratified biochemical recurrence‐free^a^ (A), distant metastasis‐free (B), and overall survival (C) in favorable‐intermediate risk patients. Study outcomes were modeled as time‐to‐event endpoints using race‐stratified Kaplan–Meier estimation curves. Low and favorable‐intermediate risk strata were defined by National Comprehensive Cancer Network criteria.^21^ AA, African American; CA, Caucasian American; BCR, biochemical recurrence; DM, distant metastasis; OS, overall survival. ^a^The 15‐year estimate cannot be provided among Favorable‐Intermediate due to no BCR events among AA men at this time point

Race‐stratified KM estimation curves (Figure 1A–C) revealed no statistically significant difference in 5‐, 10‐, and 15‐year BRFS, DMFS, or OS for low‐risk AA versus CA patients. Similarly, no statistically significant differences were seen in race‐stratified KM estimation curves 5‐, 10‐, and 15‐year BRFS, DMFS, or OS for favorable‐intermediate risk AA versus CA patients (Figure 2A–C).

Multivariable Cox PH analysis was performed for each of the study endpoints (Table 2). Factors predictive of poorer BRFS included: greater prostate volume (≥48.07 cc) (HR = 4.2, 95% CI = 1.3–13.9, *p* = 0.02), PSA nadir ≥ 0.2 ng/ml (HR = 4.3, 95% CI = 1.7–10.6, *p* = 0.002), and faster TTN (<2.3 years) (HR = 4.9, 95% CI = 2.3–10.5, *p* < 0.001). However, no significant difference in BRFS was observed across race. Factors that were predictive of poorer DMFS in the Cox PH model included: favorable‐intermediate versus low‐risk disease (HR = 4.2, 95% CI = 1.1–16.3, *p* = 0.04) and PSADT < 10 months (HR = 33.9, 95% CI = 4.1–277.5, *p* = 0.001). Again, race was not predictive of DMFS. Finally, Cox PH analysis revealed poorer OS for: increasing age (HR = 1.05, 95% CI = 1.02–1.08, *p* = 0.001), favorable‐intermediate versus low‐risk disease (HR = 1.6, 95% CI = 1.1–2.4, *p* = 0.016), TTN < 2.3 years vs. ≥2.3 years (HR = 1.7, 95% CI = 1.2–2.5, *p* = 0.004), and presence versus absence of any major comorbidity (HR = 2.1, 95% CI = 1.4–3.1, *p* < 0.001).

**TABLE 2:** Multivariable Cox Proportional Hazards Analysis Models predicting Biochemical recurrence‐free survival, Distant metastasis‐free survival, and Overall survival following EBRT treatment (*n* = 405^a^ ^–^ ^c^)

Patient characteristic	Biochemical recurrence‐free survival (BRFS)		Distant metastasis‐free survival (DMFS)		Overall survival (OS)	
	HR (95% CI)	*p*‐value	HR (95% CI)	*p*‐value	HR (95% CI)	*p*‐value
Age at EBRT (years)	1.01 (0.96, 1.06)	0.84	0.98 (0.9, 1.1)	0.64	1.05 (1.02, 1.08)	**0.001**
Prostate volume^b^ (cubic centimeters, cc)			NA	NA	NA	NA
28.4–36.4 vs. <28.4	1.8 (0.6, 5.8)	0.31				
36.5–48.06 vs. <28.4	2.5 (0.8, 7.8)	0.13				
≥48.07 vs. <28.4	4.2 (1.3, 13.9)	**0.02**				
Self‐reported race						
African American vs. Caucasian American	1.3 (0.6, 2.7)	0.58	2.3 (0.6, 9)	0.24	1.3 (0.8, 2)	0.29
NCCN risk stratum						
Favorable‐Intermediate vs. Low	1.1 (0.5, 2.3)	0.84	4.2 (1.1, 16.3)	**0.04**	1.6 (1.1, 2.4)	**0.016**
PSA Nadir (ng/ml)						
≥0.2 vs. <0.2	4.3 (1.7, 10.6)	**0.002**	1.6 (0.3, 8.5)	0.56	1.1 (0.7, 1.6)	0.82
Time to PSA nadir^c^ (years)						
<2.3 vs. ≤2.3	4.9 (2.3, 10.5)	**<0.001**	0.8 (0.2, 3.8)	0.76	1.7 (1.2, 2.5)	**0.004**
PSADT (months)						
≥10 vs. No BCR	NA	NA	2.8 (0.6, 13.9)	0.20	NA	NA
<10 vs. No BCR			33.9 (4.1, 277.5)	**0.001**		
Major comorbidities						
Yes vs. No					2.1 (1.4, 3.1)	**<0.001**

^a^ Exclusion of study subjects was made for the following reasons: receipt of secondary HT (*n* = 68), missing PSA nadir or missing prostate volume information (*n* = 33).

^b^ The model was also adjusted for primary treatment year, primary treatment type, and primary treatment technique.

^c^ Time to PSA nadir was categorized using the median split for time from RT to PSA nadir (in years).

Bold indicates significant values of *p* < 0.05.

Due to sporadic missing data, the effective sample size in the Cox PH models decreased from *n* = 840 to *n* = 405. Therefore, a detailed comparison of descriptive characteristics was conducted to ensure comparability in distributions of key patient characteristics across the overall cohort (*n* = 840) versus the patient subset retained in the Cox PH models (*n* = 405). No differences were observed across the groups (Table 3).

**TABLE 3:** Characteristics of the Overall Study Cohort (*N* = 840) compared to study subjects in Cox Proportional Hazards (PH) models (*n* = 405)

	Overall study cohort *N* = 840	Study subjects in Cox PH models *n* = 405
	*N* (%)	*N* (%)
Age at RT (year)		
Median (IQR^a^)	69.9 (63.8, 74.9)	69.3 (63.6, 75.1)
Follow‐up Time after EBRT, (year)		
Median (IQR)	6.5 (4.3, 10.9)	5.9 (4.1, 9.1)
Time from RT to PSA Nadir^b^ (year)		
Median (IQR)	2.3 (1.3, 4.3)	2.5 (1.4, 4.3)
Time from RT to BCR, (year)		
Median (IQR)	5.3 (3.4, 7.8)	6.2 (4, 8)
Time from EBRT to Mets, (year)		
Median (IQR)	8.1 (3.1, 11)	8.4 (4.2, 11)
Dosage(cGy)		
Median (IQR) for CT based/3DCRT	7000 (6840, 7040)	7000 (6840, 7200)
Median (IQR) for IMRT	7800 (7600, 7800)	7800 (7600, 7800)
Prostate volume, (cc)		
Median (IQR)	36.5 (28.4, 48.1)	36.5 (28.2, 49.5)
Biochemical recurrence (BCR)^c^, *N* (%)	151 (18.7)	37 (9.1)
Distant metastasis, *N* (%)	29 (3.5)	11 (2.7)
Overall death, *N* (%)	333 (39.6)	125 (30.9)
Race		
AA	268 (32)	131 (32)
CA	572 (68)	274 (68)
Primary treatment type, *N* (%)		
EBRT alone	748 (89)	360 (89)
EBRT with HT^d^	92 (11)	45 (11)
Primary treatment technique, *N* (%)		
CT based/3D CRT	530 (63.1)	224 (55.3)
IMRT	310 (36.9)	181 (44.7)
Secondary HT^e^, *N* (%)		
Time from RT to secondary HT (year) Median (IQR)	6.2 (3.8, 9.4)	–
No	772 (91.9)	405 (100)
Yes	68 (8.1)	–
PSA nadir (ng/ml), *N* (%)		
Median (IQR)		
<0.2 (undetectable)	241 (29.9)	130 (32)
≥0.2 (detectable)	566 (70.1)	275 (68)
Missing/Unknown	33	–
PSA at diagnosis (ng/ml), *N* (%)		
Median (IQR)	5.9 (4.3, 8.3)	
<4	173 (20.6)	87 (21.5)
4–10	541 (64.4)	261 (64.4)
10–20	126 (15)	57 (14.1)
PSADT^g^ (months), *N* (%)		
No BCR	656 (81.3)	368 (90.9)
<10	52 (12.3)	29 (7.1)
≥10	99 (6.4)	8 (2)
Missing/Unknown	33	–
Clinical T stage, *N* (%)		
≤T2a	758 (90.2)	371 (91.6)
T2b–T2c	82 (9.8)	34 (8.4)
Biopsy Gleason score, *N* (%)		
≤6	691 (82.3)	324 (80)
3 + 4	149 (17.7)	81 (20)
Obesity (BMI ≥ 30.0 kg/m^2^), *N* (%)		
No	535 (73.5)	264 (71.7)
Yes	193 (26.5)	104 (28.3)
Missing/Unknown	112	37
Major Comorbidities^f^, *N* (%)		
No	519 (61.8)	255 (63)
Yes	321 (38.2)	150 (37)

^a^ IQR, interquartile range.

^b^ PSA nadir was defined as the lowest absolute PSA value following EBRT treatment *and* prior to secondary HT, if applicable.

^c^ BCR = biochemical recurrence was defined as a rise in PSA ≥ 2 ng/ml above the nadir PSA value.

^d^ HT, hormone treatment defined as any HT within 9 months prior to *or* 1 year following EBRT treatment (start date).

^e^ Secondary HT was defined as HT >1 year following EBRT and before, distant metastasis.

^f^ Any major comorbidity included: chronic obstructive pulmonary disease (COPD), cardiovascular disease (CVD), Cerebral Vascular Accident (CVA), and/or Other Cancer.

^g^ PSADT, PSA doubling time was calculated among those who experienced a BCR event (*n* = 151) using all PSA values within 2 years after BCR, censored at the time of metastasis or use of secondary HT. PSADT was then computed as the natural logarithm of 2 divided by the slope obtained from fitting a linear regression of the natural log(PSA)/time.

Time trends in median EBRT doses for patients undergoing 3D CRT (*n* = 530) and IMRT (*n* = 310) were also examined. These trends were examined for the overall study cohort, as well as stratified into race by biopsy grade subgroups (i.e., CA/3 + 3, AA/3 + 3, CA/3 + 4, and AA/3 + 4). Though overall increases in radiation dose were observed over time, particularly between 1997 and 2004, these increases were comparable in all race by grade categories. As expected, 3D CRT was the most common mode of EBRT delivery until 2003, largely replaced by IMRT in 2004 (*data available upon request*).

For a subset of patients, underlying cause of death was examined, using data obtained from the CDC National Death Index Office among the 333 subjects who died in the study cohort. Using these data, an attempt was made to construct a prediction model for prostate cancer‐specific mortality (PCSM) estimates, with race as a key independent variable. However, due to the very small number of prostate cancer‐specific deaths (*n* = 20, 2.4%), and a median time from EBRT to PCSM of 10.8 years, effect estimates were unreliable and are not presented. However, a KM analysis of the 20‐year cumulative risk of PCSM demonstrated no racial differences in PCSM (log‐rank *P* = 0.76; *data available upon request*).

## 4 DISCUSSION

This study confirmed that, in a cohort with equal healthcare access, enrolled over a quarter of a century, no racial differences were noted in multiple PCa outcomes for patients with low and favorable‐intermediate risk disease who were treated with EBRT. In following this cohort over an extended period, this study accounted for notable changes in radiation delivery over time.

In contrast to the present study, findings from the Surveillance, Epidemiology, and End Results (SEER)‐Medicare populations have shown the age‐adjusted PCa mortality rates to be twice as high for AA compared to CA men.^25^ However, recent reports on the history of PCa indicate that AA men have had a significantly higher incidence of preclinical disease, greater risk of metastatic progression, lower likelihood of receiving treatment with curative intent, and higher risk of PCSM.^26^, ^27^ Furthermore, it has been suggested that AA men have higher grade and more advanced disease at presentation. These risk factors have been associated with worse cancer control outcomes for AA men compared to CA men.^4^, ^7^, ^28^, ^29^, ^30^ Williams et al. evaluated over 7300 men demonstrating significant differences in PCa outcomes among non‐Hispanic AA and white men, and found that AA men experienced higher PCSM than CA men.^31^

Conversely, our study supports previous work showing little or no racial differences in PCa outcomes following treatment.^32^, ^33^, ^34^, ^35^, ^36^, ^37^, ^38^ In a simulation study conducted on the National Cancer Database (NCDB) which captures roughly 60% of all PCa cases diagnosed in the US, no significant racial differences in survival (AA compared to CA men) with simulated equal access to healthcare, and better survival outcomes for AA men were noted after simulating equal treatment and tumor characteristics.^39^ In other studies that have examined the U.S. Department of Defense tumor registry, including within the Veteran Health Administration systems, comparable PCSM outcomes were reported for AA and CA men in these equal access healthcare systems as well.^32^, ^33^, ^34^, ^35^, ^36^, ^37^

During this extended study period, the types and approaches to EBRT delivery evolved with unknown impact on race‐specific outcomes, as well as access to care and treatment intensity.^8^, ^9^, ^10^, ^11^ For example, with the introduction of IMRT, barriers to access of care arose, including geographical location and cost‐inhibitive technological incorporation, resulting in more CA than AA men receiving IMRT in the years following its introduction.^8^, ^9^ However, in this study, there were no notable differences in IMRT initiation and no significant differences in long‐term outcomes between racial groups before or after the years when use of IMRT became widespread. Previous research has indicated that AA men experience longer delays to the start of treatment and less aggressive treatment plans, a finding that was not observed in this study.^11^, ^40^, ^41^, ^42^ It has been shown that AA men are less likely to receive definitive treatment, and that health insurance coverage is associated with a reduction in racial disparity.^43^, ^44^, ^45^ This study focused on a cohort diagnosed and treated within an equal access healthcare system for which all patients had insurance coverage.

Despite our findings of comparable oncologic outcomes, AA men in this study cohort presented with higher Gleason score and were diagnosed at younger ages. Yet, following diagnosis, they experienced the same intensity of treatment as indicated by median EBRT dosage and receipt of adjuvant and/or salvage HT, and ultimately experienced no difference in long‐term outcomes compared to CA men. Similar findings were shown by Shah et al., demonstrating AA men presented at younger ages and with a higher Gleason Score, and yet such baseline differences did not translate into poorer OS, BRFS, or DMFS compared to CA men.^46^ Moreover, in provocative findings from nearly 6000 patients in RTOG trials, AA men appeared to have more radiosensitive tumors and more robust immunologic responses to radiation treatment, resulting in improved outcomes, compared to CA men.^47^

The current study also found several important clinic‐pathological factors associated with poorer BRFS, including larger prostate volume (≥48.07 cc), failing to achieve PSA nadir, and time to PSA nadir < 2.3 years. None of these variables differed across race. Similar to our study, Kaminski et al. found that in men who received 3D CRT for treatment of “favorable” PCa (defined as having a PSA < 10 ng/ml, Gleason score 2–6, and T1–T2a disease), prostate volume was a significant predictor of biochemical progression (*p* = 0.04).^48^ While the observation that a shorter TTN was strongly associated with poorer BRFS and OS was unexpected, this has been reported previously.^49^, ^50^ Explanations for this counterintuitive finding have included more aggressive disease and/or lead time bias among those with shorter TTN's, with more frequent patient follow‐up in such patients, to ensure optimal outcomes.

### 4.1 Limitations

Potential limitations include that this study utilized a military population, with the exception of one site, which may differ from civilian populations. However, this allowed for assessment of an equal access healthcare system enriched with a large percentage of AA men. The 27‐year study period was advantageous for examining disease progression as EBRT evolved, but also posed challenges due to changes in the delivery of EBRT. To address these concerns, this study analyzed the impact of several critical treatment‐related factors including median EBRT dose, EBRT type, and biopsy grade to ensure comparability across race over time. Despite the extended study period, the median follow‐up time was 6.5 years, making it a challenge to examine longer‐term endpoints. However, there were a robust number of BCR events and deaths (*all cause*) that were shown to be comparable across race, with detailed clinical variable adjustment. Moreover, these endpoints are challenging for any study focused on prognostication of aggressive disease for patients with low and favorable‐intermediate risk PCa. Though we could not model PCSM in this study, we were able to examine DMFS, a strong correlate of PCSM, and our analysis revealed no racial differences, albeit with a small number of events. Lastly, race was assessed by patient self‐report, which may not represent true genetic differences. However, a large, racially/ethnically diverse study of data from “23andMe”––a company that has compiled a global database of over 5 million customers, with roughly 80% sharing genotypic and phenotypic information for research on human ancestry and diseases^51^––indicate that self‐report is a fairly accurate indicator of genetic origin, especially for AA and CA men.^52^ Future research should examine PCSM and DMFS in larger cohorts with underrepresented racial/ethnic groups.

## 5 CONCLUSION

In contrast to many studies that support poorer PCa outcomes for AA compared to CA men, this study found no associations between race and cancer progression or overall survival among patients undergoing EBRT as primary treatment for low and favorable‐intermediate risk PCa. The healthcare setting from which patients were diagnosed and treated for over a quarter of a century is likely a key factor contributing to this absence of racial disparities. Overall, the current study suggests that, within an equal access healthcare system, racial disparities in PCa outcomes can be reduced, at least among low and favorable‐intermediate risk patients treated with EBRT.

## CONFLICT OF INTEREST

The authors declare no potential conflict of interest.

## AUTHOR CONTRIBUTIONS

Sean P. Stroup: Conceptualization, project administration, supervision, writing—original draft, and writing—review and editing. Audry H. Robertson: Investigation, writing—original draft and writing—review and editing. Kayla C. Onofaro: Investigation, writing—original draft and writing—review and editing. Michael G. Santomauro: Conceptualization and writing—review and editing. Nicholas R. Rocco: Conceptualization, and writing—review and editing. Huai‐ching Kuo: Data curation, formal analysis, methodology, software, visualization, and writing—review and editing. Avinash R. Chaurasia: Writing—review and editing. Samantha Streicher: Writing—review and editing. Darryl Nousome: Writing—review and editing. Timothy C. Brand: Project administration, supervision, and writing—review and editing. John E. Musser: Project administration, supervision, and writing—review and editing. Christopher R. Porter: Project administration, supervision, and writing—review and editing. Inger L. Rosner: Project administration, supervision, and writing—review and editing. Gregory T. Chesnut: Supervision and writing—review and editing. Anthony D'Amico: Conceptualization and writing—review and editing. Grace Lu‐Yao: Writing—review and editing Jennifer Cullen: Conceptualization, funding acquisition, methodology, project administration, supervision, writing—original draft, and writing—review and editing.

## DISCLAIMERS

The contents of this publication are the sole responsibility of the authors and do not necessarily reflect the views, opinions, or policies of Uniformed Services University of the Health Sciences (USUHS), the Henry M. Jackson Foundation for the Advancement of Military Medicine, Inc., the Department of Defense (DoD) or the Departments of the Army, Navy, or Air Force. Mention of tradenames, commercial products, or organizations does not imply endorsement by the U.S. Government.

## Data Availability

The data supporting the findings of this study are available from the Center for Prostate Disease Research (CPDR). Restrictions and additional conditions may apply to the availability of these data from the Defense Health Agency (DHA).
